# Applicability of methylliberine as a salivary marker under postprandial conditions

**DOI:** 10.1016/j.ijpx.2026.100584

**Published:** 2026-06-15

**Authors:** Toni Wildgrube, Nikolaus Alexander Link, Benno Ritucci, Michael Grimm, Stefan Engeli, Werner Weitschies, Philipp Schick

**Affiliations:** a Department of Biopharmaceutics and Pharmaceutical Technology, Center of Drug Absorption and Transport (C_DAT), University of Greifswald, Felix-Hausdorff-Str. 3, 17487 Greifswald, Germany; b Institute of Pharmacology, Center of Drug Absorption and Transport (C_DAT), University Medicine Greifswald, Felix-Hausdorff-Str. 3, 17487 Greifswald, Germany

**Keywords:** *In vivo* study, Fed state, Caffeine, Methylliberine, Food effect, Salivary analysis

## Abstract

Salivary pharmacokinetics provide a non-invasive alternative to blood sampling and are widely applied for the investigation of formulation performance and physiological processes such as gastric emptying. In this context, caffeine is an established salivary marker due to its robust and well characterised pharmacokinetic behaviour and low susceptibility to postprandial physiological changes, allowing its use under both fasted and fed conditions. Recently, methylliberine has been introduced as a novel salivary marker and shown to be suitable for the assessment of gastric emptying under fasted conditions, offering advantages such as a short elimination half-life and simplified study designs as caffeine abstinence is unnecessary. However, its applicability as a salivary marker under postprandial conditions has not yet been evaluated. The present study aimed to assess whether methylliberine can be applied as a salivary marker under fed state conditions in a manner comparable to caffeine. Compression-coated tablets containing 100 mg methylliberine and 25 mg caffeine were developed to prevent oral contamination and characterised *in vitro*. In a randomised three-way crossover study, twelve healthy volunteers received the formulation in the fasted state, after a standardized light meal and a high fat meal. Saliva samples were collected for 24 h and analysed by LC-MS/MS. Both analytes were rapidly detectable in saliva after administration. Postprandial dosing resulted in delayed t_max_ values for both substances, consistent with altered gastric emptying. While systemic exposure of caffeine remained unaffected by food intake, methylliberine exhibited a calorie-dependent reduction in exposure, which was statistically significant after the high-fat meal compared with fasted administration. These findings indicate that methylliberine might have limitations as a salivary marker under postprandial conditions. At the same time, its sensitivity to the physiology of the fed state identifies methylliberine as a potential substance for investigating the mechanisms of food effects.

## Graphical abstract

**Figure:**
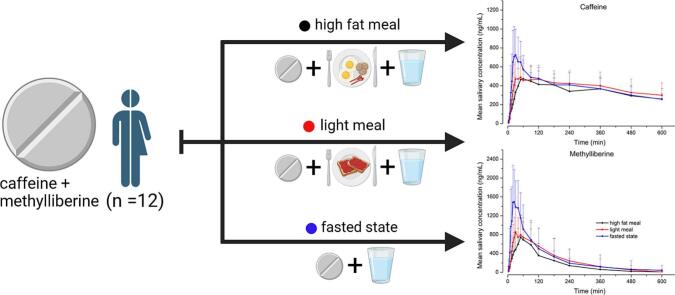

## 1 Introduction

A deeper understanding of gastrointestinal physiology and its impact on oral drug absorption requires the application of suitable *in vivo* investigation techniques (Schwizer et al., 2006; Ajaj, 2004; Cappello et al., 2000; Kelly et al., 2003; Koziolek et al., 2015a). Salivary pharmacokinetics have emerged as an increasingly important tool for the non-invasive investigation of the performance of oral drug formulations as well as key gastrointestinal physiological processes such as gastric emptying (Sager et al., 2018; Grimm et al., 2023a; Senekowitsch et al., 2022; Grimm et al., 2023b). In contrast to conventional plasma based pharmacokinetic studies, saliva sampling enables repeated measurements without invasive blood withdrawal or the need for specially trained personnel, thereby facilitating simplified and more participant friendly study designs (Hofman, 2001; Michalke et al., 2015; Caporossi et al., 2010). For selected compounds, it has been demonstrated that concentrations in saliva accurately reflect plasma levels and changes in systemic exposure (Newton et al., 1981; Zylber-Katz et al., 1984; Glynn and Bastain, 2011; Senekowitsch et al., 2026). Substances that meet these criteria can be considered as salivary markers. The BCS-class I compound caffeine is among the most widely used salivary marker and has been successfully applied in numerous studies to investigate both gastric emptying and the behaviour of oral dosage forms under fasted and postprandial conditions (Sager et al., 2018; Grimm et al., 2023a; Tzakri et al., 2023; Senekowitsch et al., 2023). Its suitability is largely attributed to rapid absorption, predictable pharmacokinetics, and a well-established safety and tolerability profile in healthy volunteers, as well as a high robustness towards postprandial physiological changes. This makes caffeine a highly effective marker that also can be used independently of the prandial state. (Scientific Opinion on the Safety of Caffeine, 2015). However, the ubiquitous presence of caffeine in the daily diet represents a practical limitation, as extended abstinence periods or the use of costly, isotopically labelled compounds are often required to control baseline concentrations, substantially complicating study logistics (Grimm et al., 2023c).

More recently, methylliberine has been introduced as a novel salivary marker and has already been successfully applied for the assessment of gastric emptying under fasted conditions (Wildgrube et al., 2025). In comparison with caffeine, methylliberine offers a number of practical advantages, including a shorter elimination half-life and the absence of relevant endogenous baseline concentrations due to its rare dietary occurrence, thereby enabling simplified study designs without extensive abstinence requirements (Mondal et al., 2022; Wang et al., 2020). While the applicability of methylliberine as a salivary marker in the fasted state has thus been demonstrated, it has not yet been investigated whether these properties are maintained under postprandial conditions. In order to facilitate the broader application of salivary tracer technology (STT) beyond the fasted state, it is essential that methylliberine itself does not exhibit pronounced prandial dependent behaviour. This would otherwise complicate the interpretation of physiological or formulation related effects.

It is well established that food intake can induce significant changes in gastrointestinal physiology. These changes include modulation of gastric emptying, luminal composition, bile salt concentrations, intestinal fluid volumes and splanchnic blood flow (Koziolek et al., 2019; Deng et al., 2017; Kambayashi and Shirasaka, 2023; Koziolek et al., 2015b; Koziolek et al., 2016; Fleisher et al., 1999; Hunt and Stubbs, 1975). All of these factors may influence the absorption and systemic exposure of orally administered compounds. In accordance with the clinical relevance of these physiological alterations, O'Shea et al. reported that more than 40% of orally administered drugs approved by the EMA and FDA since 2010 exhibit clinically relevant food interactions, thereby highlighting the high practical importance of prandial influences on drug exposure (O'Shea et al., 2019). Food related changes in drug exposure are commonly referred to as food effects. The investigation of food effects has become an important part of oral drug development, as both positive and negative food effects may influence bioavailability and therapeutic efficacy (Abuhelwa et al., 2017; Singh, 1999).

Such food effects may arise from both specific and non-specific mechanisms. It is important to note that the effects of food are substance-specific, even in cases where the underlying mechanisms are not yet fully understood. An absence of mechanistic understanding does not necessarily imply non-specific food changes, as otherwise comparable effects would be expected for all orally administered compounds. Rather, food-related changes in exposure typically arise from the interplay between a compound's physicochemical properties, its absorption characteristics, and food-induced physiological alterations.

Against this background, the present study aimed to evaluate the applicability of methylliberine as a salivary marker under postprandial conditions. Specifically, we investigated whether methylliberine itself exhibits prandial dependent changes in systemic exposure (food effect) and to what extent its postprandial behaviour differs from that of the established marker caffeine. Methylliberine and caffeine were administered simultaneously as compression-coated tablets to prevent oral contamination. The pharmacokinetic parameters were evaluated under three different conditions: fasted, following a standardized high-fat meal, and after a standardized light meal.

## 2 Materials and Methods

### 2.1 Compression Coated Tablets

For the production of the tablets used in the present study, caffeine and amorphous colloidal silicon dioxide (Aerosil®) were obtained from Fagron GmbH & Co. KG (Glinde, Germany), methylliberine was sourced from Nootropics Depot (Grand Rapids, Michigan, USA). Saccharin sodium, hydroxypropyl methylcellulose (Methocel® E4M), magnesium stearate and black iron oxide were all ordered from Caesar & Loretz GmbH (Hilden, Germany). Croscarmellose sodium (Vivasol®) and silicified microcrystalline cellulose (Prosolv® SMCC 90) were obtained from JRS Pharma GmbH & Co. KG (Barsbüttel, Germany). Microcrystalline cellulose (Avicel® PH 102) was obtained from F. Hoffmann-La Roche AG (Basel, Switzerland). The aluminium pouches used to seal the single tablets were obtained from Ströbel GmbH (Langenzenn, Germany). The preparation and composition of the compression-coated tablets employed in this study were based on a previously established formulation by Tzakri et al. *(*Tzakri et al., *2023**)*. The production of the compression-coated tablets was carried out in a two-step process. In the first step, the powder mixture for the tablet cores was manually compressed into round flat cores with a diameter of 7 mm using an eccentric tablet press Nagema KP2 (VEB Kombinat Nagema, Dresden, Germany). Each tablet core was formulated to contain 25 mg of caffeine and 100 mg of methylliberine. In the second step, the core tablets were placed in a 10 mm biconvex die and the powder mixture intended for the outer layer was compressed to obtain the final compressed tablets. The quantitative details of the excipients and active substances used for the fabrication of both the tablet core and the compression coating are presented in Table 1, Table 2, respectively. Following batch production, the tablets were individually packaged in aluminium pouches and heat-sealed.

**Table 1:** Composition of the tablet cores (theoretically calculated masses and percentages per unit).

Ingredients	Quantity (mg)	Quantity (%)
Methylliberine	100.0	58.8
Caffeine	25.0	14.7
Saccharine sodium	10.0	5.9
Avicel® PH 102	18.0	10.6
Vivasol®	8.5	5.0
Black iron oxide	3.4	2.0
Aerosil®	3.4	2.0
Magnesium stearate	1.7	1.0
Total	170.0	100

**Table 2:** Composition of the outer layer (theoretically calculated masses and percentages per unit).

Ingredients	Quantity (mg)	Quantity (%)
Prosolv® SMCC HD 90	207.74	94.0
Methocel® E4M	8.84	4.0
Magnesium stearate	4.42	2.0
Total	221.0	100

Quality control of both the core and the compression-coated tablets was performed. Content uniformity Ph.Eur. 2.9.40 (*n* = 10), hardness (*n* = 6), height (*n* = 6) Ph.Eur. 2.9.8, friability Ph.Eur. 2.9.7, disintegration time Ph. Eur. 2.9.1 (*n* = 6), dissolution (*n* = 3), and stability testing of formulations, including long-term stability: 25 °C and 60% relative humidity (*n* = 3) and accelerated stability: 40 °C and 75% relative humidity (*n* = 3), were investigated. All analyses were carried out in accordance with the procedures described in the current edition of the European Pharmacopoeia (Ph. Eur. 11.0).

*In vitro* dissolution experiments were performed according to the pharmacopeial USP dissolution method using a pharmacopeial USP 2 Apparatus (PT-DT8 USP II, Pharma Test Apparatebau AG, Hainburg, Germany). Dissolution studies were conducted under worst case conditions in 300 mL simulated gastric fluid without pepsin (SGFsp, pH 1.2) at a controlled temperature of 25 °C and a paddle rotation speed of 75 rpm. The dissolution experiments were conducted over a total period of 10 min.

Samples of 1 mL were withdrawn at 30 s intervals, and the withdrawn volume was immediately replaced with an equal volume of fresh SGFsp to ensure constant dissolution conditions. In order to prevent coning of the tablet components, Apex (peak) vessels (Agilent, Santa Clara, USA) were used throughout of the experiments. The concentrations of methylliberine and caffeine in the collected samples were determined using a previously published and validated HPLC-UV/Vis method (Wildgrube et al., 2025). Dissolution experiments were performed in triplicate (*n* = 3) for both core and compression-coated tablets.

### 2.2 *In vivo* Study

The *in vivo* study investigated the influence of different prandial states (A: high fat meal, B: light meal, and C: fasting state) on the pharmacokinetic behaviour of methylliberine. The Salivary Tracer Technique (STT), as described by Sager et al., was utilised for the collection of samples. This technique has been established as a non-invasive method for the independent and reliable collection of saliva samples (Sager et al., 2018).

The three-arm *in vivo* study was conducted as a randomised crossover design with twelve young, healthy subjects (six women and six men). The sequence of the study arms was determined by means of block randomisation resulting in different treatment sequences for the participants (A-B-C, B-C-A, or C-A—B). The individual study periods were conducted on separate study days and were separated by washout intervals of at least 72 h to minimise potential carry-over effects and residual caffeine concentrations from the previous study period. The study was conducted in accordance with the current EMA (European Medicines Agency) and FDA (Food and Drug Administration) guidelines for food-effect and bioequivalence studies (FDA, 2002; EMA, 2012). All study procedures further adhered to the principles of the Declaration of Helsinki (2024, Helsinki, Finland). All study participants were insured against potential risks during the study. The Ethics Committee of the University Medicine Greifswald approved the study protocol and all associated documents (internal registration number: BB 175/24). The study has also been registered in the German Clinical Trials Register (Deutsches Register Klinischer Studien) under the ID DRKS00035516. The study protocol stipulated a 72-h abstinence from caffeine before each study day to minimise basal physiological caffeine concentrations in saliva. Participants were additionally instructed to abstain from alcohol consumption for at least 48 h before each study day. Additionally, participants were instructed to abstain from solid food for 10 h prior to the start of the study. Furthermore, volunteers were instructed to abstain from consuming caloric beverages a minimum of 3 h prior to the beginning of the study. However, they were permitted to consume non caloric liquids up to 90 min before. In study arms A and B, participants rinsed their mouth with water after meal consumption and before administration of the study formulation in order to minimise potential contamination of subsequent saliva samples by food residues. The study was carried out in the Clinical Research Unit of the Centre of Drug Absorption and Transport (C_DAT) in Greifswald. Regardless of the designated study arm, all participants were obliged to ingest a compression coated tablet with 240 mL of tap water (Stadtwerke Greifswald, Germany).

The main difference between the individual study arms was the prandial state of the participants immediately prior to tablet administration. In study arm A, participants received a high fat, high caloric standard meal (approximately 1000 kcal) before taking the tablets. In study arm B, they consumed a light meal (approximately 500 kcal). In study arm C, the participants remained in the fasted state. The specific food products and amounts used are shown in Table 3, Table 4.

**Table 3:** Composition of the high fat meal (study arm A).

Product (amount)	Name of product and manufacturer
Egg (2 pieces)	Freilandeier Eier Größe L (Poseritzer EierHOF, Germany)
Toast (2 slices)	Sammy's Super Sandwich (HARRY-BROT GmbH, Germany)
Bacon (2 strips)	Tulip Bacon (TULIP FOOD COMPANY, Denmark)
Hash brown potatoes (3 pieces)	Gut & Günstig Rösti-Ecken (Gut&Günstig Eigenmarke der EDEKA Group)
Milk (240 mL)	Gut & Günstig H-Vollmilch 3.5% Fett (Gut&Günstig Eigenmarke der EDEKA Group)
Butter (17 g)	Meggle Alpenbutter (MEGGLE AG, Germany)

**Table 4:** Composition of the light meal (study arm B).

Product (amount)	Name of product and manufacturer
Butter (15 g)	Meggle Alpenbutter (MEGGLE AG, Germany)
Strawberry yoghurt (150 g)	Ja! Fettarmer Joghurt Erdbeere (T.M.A Handelsgesellschaft GmbH, Germany)
Orange juice (200 mL)	VALENSINA Orange ohne Fruchtfleisch (VALENSINA GmbH, Mönchengladbach, Germany)
Toast (2 slices)	Sammy's Super Sandwich (HARRY-BROT GmbH, Germany)
Strawberry jam (30 g)	SCHWARTAU Extra Erdbeere (SCHWARTAUER WERKE GmbH & Co. KG, Bad Schwartau, Germany)

Saliva samples were collected self-reliantly by the participants using 2 mL SafeSeal microtubes (SARSTEDT, Nümbrecht, Germany). Unstimulated whole saliva was collected without the use of stimulatory devices. Participants were instructed to spit directly into the collection tubes and to obtain a minimum sample volume of approximately 1 mL per sampling point. To minimise potential dilution effects, fluid intake was scheduled with temporal distance from saliva collection. Participants who collected samples outside the Clinical Research Unit were likewise instructed to maintain an interval between food or beverage intake and the subsequent saliva sampling. The first sample was obtained 5 min prior to the start of each study day, in order to detect elevated basal caffeine concentrations. In study arm C (fasted state), the administration of the compression coated tablet was followed immediately by the ingestion of 240 mL of water. In the postprandial arms (A and B), participants first consumed either the high-fat meal or the light meal, respectively, and both meals were required to be eaten within 15 min. The study formulation was administered with 240 mL of water 30 min after the start of the meal in both postprandial study arms. Following administration, all study arms adhered to an identical sampling timeline. Saliva samples were collected at predetermined time points over a 24 h period, with samples taken at 4, 8, 12, 16, 20, 25, 30, 35, 40, 50, 60, 90, 120, 180, 240, 360, 480, 600 and 1440 min after the administration. At 120 and 240 min, participants were instructed to consume additional 240 mL of water. Participants in fasted study arm C were permitted to resume normal food and fluid intake after 240 min, whereas those in postprandial arms A and B were instructed to wait until 360 min. In all study arms, caffeine abstinence had to be maintained until the end of the study day (1440 min). Saliva samples at 360, 480, 600, and 1440 min could be collected independently by the volunteers outside the Clinical Research Unit. Participants were instructed to store these samples in their personal refrigerators until the subsequent study day, at which point they were to be returned to the study staff. All study samples were then stored at −80 °C (U410 Premium, Eppendorf, Germany) until analysis.

### 2.3 Sample Preparation and Analysis

The methodology for sample processing and analysis was based on a previously validated procedure described in an earlier study and was validated in accordance with EMA guideline on bioanalytical method validation (Wildgrube et al., 2025; European Medicines Agency—Committee for Human Medicinal Products (CHMP), 2011). On the day of analysis, saliva samples were thawed at room temperature and subjected to centrifugation for 10 min at 13,000 rpm (16,060 ×*g*) using a Biofuge pico centrifuge (Heraeus, Hanau, Germany). An aliquot of 100 μL was transferred into 1.5 mL microtubes (Sarstedt, Nümbrecht, Germany) and spiked with D_9_-caffeine (4 μg/mL in acetonitrile/water (10:90 *V*/V)) as an internal standard. Protein precipitation was achieved through the addition of 200 μL of acetonitrile containing 1% formic acid. Following centrifugation for 10 min at 13,000 rpm (16,060 ×g), the processed samples were transferred into HPLC vials (VWR International GmbH, Darmstadt, Germany) and analysed using a triple-quadrupole LC-MS/MS system (LCMS-8060, Shimadzu Corporation, Kyoto, Japan). The mass spectrometric detection was conducted in positive multiple reaction monitoring (MRM) mode. The validated quantification ranges were 8–2400 ng/mL for methylliberine and 5–1500 ng/mL for caffeine (Wildgrube et al., 2025).

### 2.4 Statistics

The calculation of the primary pharmacokinetic parameters (c_max_, t_max_, AUC, and t_1/2_) for caffeine and methylliberine was based on the salivary concentration-time profiles obtained in study arms A-C. If caffeine was already detectable in the blank saliva sample of a subject, the concentration measured in the pre-dose saliva sample was subtracted from all subsequent concentrations prior to further pharmacokinetic analysis. T_max_ was defined as the time corresponding to the observed maximum salivary concentration (c_max_), whereas overall exposure (AUC) was calculated using the trapezoidal rule. Half-lives (t_0,5_) were derived from the terminal log-linear phase of the concentration time curves. The parameters were calculated individually for each participant, and mean values with standard deviations (*n* = 12) were subsequently reported. Graphical visualization of salivary concentration time profiles was performed using OriginPro 8.5.1 (OriginLab, Northampton, MA, USA). In order to assess statistically significant differences between the three study arms, it was first necessary to test all datasets for normality using GraphPad Prism 5.0 (GraphPad Software, San Diego, USA). The assessment of normality was conducted using the Shapiro-Wilk test, the D'Agostino-Pearson omnibus test, and the Kolmogorov-Smirnov test. In the case of normally distributed datasets, analysis was performed using one-way ANOVA followed by Tukey's *post hoc* test. Non-normally distributed datasets were analysed using the Friedman test with Dunn's *post hoc* test.

## 3 Results

### 3.1 Quality Control of the Formulations

**Fig. 1:**
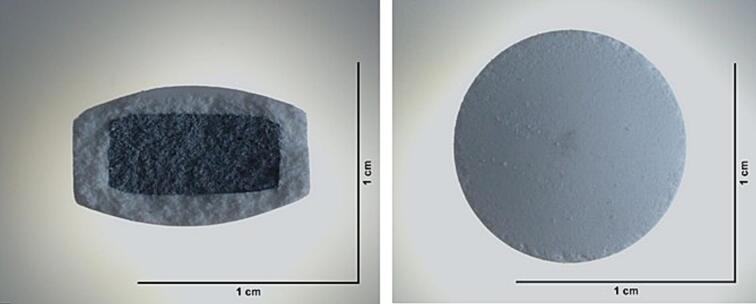
Example images of compression-coated tablets: compression-coated tablet cut in half (left); whole compression-coated tablet (right).

The compression-coated tablets were successfully manufactured. Fig. 1 shows representative images of the intact tablets as well as a formulation cut in half through the centre. The results of the physical properties of the compression-coated tablets and core tablets are shown in Table 5. Testing the mass uniformity of the tablet cores containing the active pharmaceutical ingredient demonstrated high consistency, with all six cores showing a deviation of less than 2% from their respective mean mass. The recorded mass*es* were 170 ± 1 mg for the core tablets and 412 ± 7 mg for the compression-coated tablets. Content uniformity analysis revealed caffeine content ranging from 23.5 to 26.5 mg and methylliberine content ranging from 94.4 t*o* 103.2 mg per tablet. Tablet robustness was confirmed by friability testing, resulting in a mass loss of 0.1%.

**Table 5:** Test results for evaluating the physical properties of the compression-coated tablets and core tablets (mean ± standard deviation).

Parameter	Compression-Coated Tablet	Core Tablet
Average mass [mg]	412 ± 7	170 ± 1
Height [mm]	6.2 ± 0,1	3.8 ± 0,1
Hardness [N]	86 ± 10	20 ± 2
Disintegration time	within 52 s	within 43 s

#### 3.1.1 *In vitro* Dissolution

**Fig. 2:**
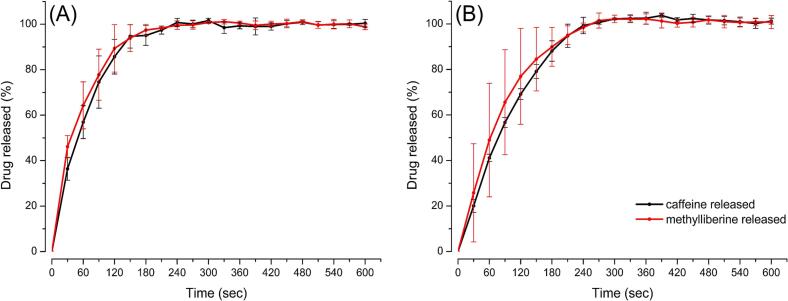
Dissolution profiles of the tablet cores (A) and compression coated tablets (B) filled with caffeine (black) and methylliberine (red) (mean of *n* = 3 ± SD). (For interpretation of the references to colour in this figure legend, the reader is referred to the web version of this article.)

Fig. 2 illustrates the dissolution profiles obtained using the paddle apparatus for both the tablet cores and the final compression-coated tablets. The formulations contained 25 mg of caffeine and 100 mg of methylliberine each. Both model drugs were released rapidly from both the tablet cores and the compression-coated tablets. Approximately 85% of caffeine and methylliberine were released from the tablet cores within 120 s. For the final compression-coated tablets, the release was slightly prolonged, with 85% release achieved after 180 s. After 240 s, complete release of both caffeine and methylliberine was observed in all cases.

**Fig. 3:**
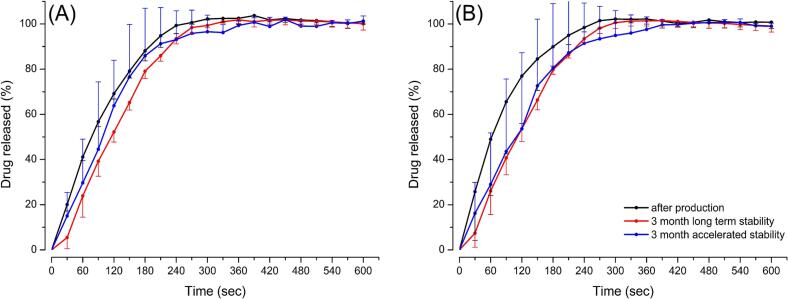
Dissolution profiles of the compression coated tablets filled with caffeine (A) and methylliberine (B) after production (black), 3-month long term stability (red) and 3 month accelerated stability (blue), (mean of *n* = 3 +/− SD). (For interpretation of the references to colour in this figure legend, the reader is referred to the web version of this article.)

The findings after three month-long term and accelerated stability study are represented in Fig. 3. In comparison with freshly prepared formulations following production, the compression-coated tablets exhibited a slightly delayed release following three months of storage under defined conditions. For both caffeine and methylliberine, the time required to reach 85% drug release increased from 180 s immediately after production to 210 s following both the three-month long term and accelerated stability studies.

### 3.2 Study Population

The demographic characteristics of the study participants are summarised in Table 6. All subjects were found to be in accordance with the inclusion and exclusion criteria specified in the study protocol. The study formulation was well tolerated by all participants, and no adverse events were reported, with all 12 volunteers successfully completing all three study days.

**Table 6:** Demographic data of the subjects (*n* = 12).

Parameter	Median (Range)	Mean ± standard deviation
Sex	m:6, f:6	–
Age	23 (19–36)	23.7 ± 1.2
Height (cm)	178 (167–200)	177.7 ± 11.0
Weight (kg)	65 (51–93)	71.5 ± 13.6
BMI (kg/m^2^)	22.1 (18.3–26.9)	22.4 ± 2.0

m – male, f – female, BMI – body mass index.

### 3.3 Study results

#### 3.3.1 Mean Profiles

**Fig. 4:**
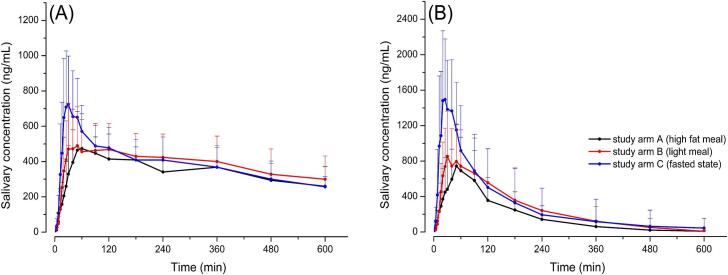
Mean salivary concentration profiles of caffeine (A) and methylliberine (B) after ingestion of a compression coated tablet containing caffeine and methylliberine under different fed and fasted conditions: after intake of a high fat meal (study arm A, black line), a light meal (study arm B, red line) or in fasted state (study arm C, blue line), (mean of *n* = 12, + SD). (For interpretation of the references to colour in this figure legend, the reader is referred to the web version of this article.)

The mean salivary profiles of caffeine and methylliberine in study arms A-C are shown in Fig. 4. Individual salivary concentration time profiles of all twelve study participants are provided in the Supplementary File (Figs. S1 and S2). Oral contamination of the oral cavity due to early disintegration of the compression-coated tablets did not occur in any of the study participants. Detectable baseline salivary caffeine levels were observed in participants 10 and 12 across all study arms, in participant 8 in study arm C, as well as in participant 9 in study arms B and C. In these cases, caffeine concentrations measured in the initial baseline saliva sample were used for baseline correction and subtracted from all subsequent measurements.

A rapid increase in the measured salivary concentrations was observed across all study arms (A-C), independent of the prandial state of the subjects. For methylliberine, a decrease in the overall exposure (AUC_0__–__1440min_) was observed as the caloric content of the administered test meal increased, whereas caffeine exposure remained almost constant across the study arms. The postprandial study arms A and B exhibited a greater t_max_ and a reduced c_max_ compared with fasted administration in study arm C for both substances. Due to the short half-life of methylliberine, complete characterization of total exposure was achieved for all study arms within the 24-h observation period. A comprehensive overview of the pharmacokinetic parameters is provided in Table 7.

**Table 7:** Overview of pharmacokinetic parameters for 25 mg caffeine and 100 mg methylliberine following fed and fasted administration using a compression coated tablet (*n* = 12, mean ± standard deviation).

Parameter	Study arm	Caffeine	Methylliberine
	High fat meal	69 ± 46	48 ± 17
t_max_ (min)	Light meal	65 ± 35	50 ± 27
	Fasted state	29 ± 12	26 ± 11
	High fat meal	534 ± 179	867 ± 397
c_max_ (ng/mL)	Light meal	596 ± 201	1033 ± 440
	Fasted state	842 ± 251	1894 ± 573
	High fat meal	5954 ± 1878	1873 ± 1133
AUC_0__–__1440_ (ng/mL*h)	Light meal	6915 ± 2813	2622 ± 1952
	Fasted state	6952 ± 1731	3362 ± 3164
	High fat meal	15.50 ± 9.22	1.34 ± 0.51
t_0,5_ (h)	Light meal	14.73 ± 6.57	1.29 ± 0.35
	Fasted state	15.56 ± 6.03	1.36 ± 0.75

### 3.4 Statistics

**Fig. 5:**
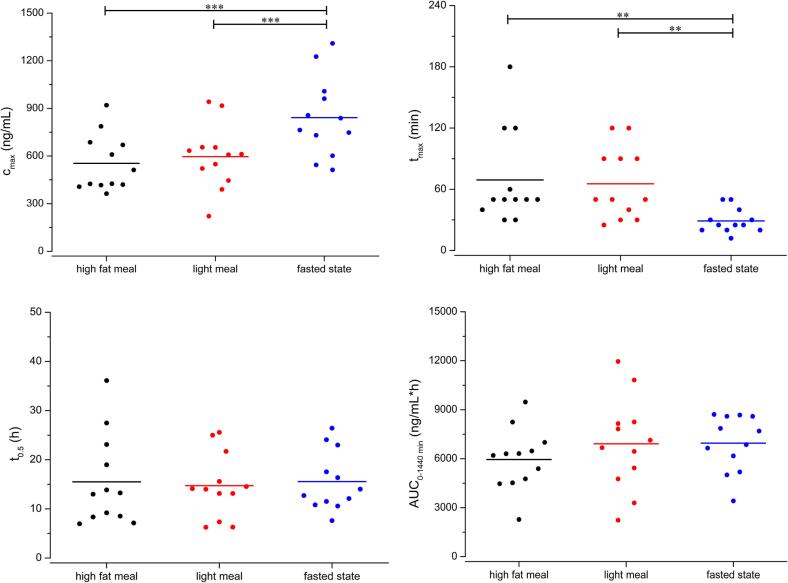
Statistical comparison of c_max_, t_max_, AUC_0__–__1440min_, and t_0.5_ for caffeine across study arms A-C. Differences in c_max_, AUC_0__–__1440min_, and t_0.5_ were assessed using ANOVA followed by Tukey's post-hoc test; t_max_ was analysed using the Friedman test with Dunn's post-hoc test. *p < 0.05; **p < 0.01; ***p < 0.001.

**Fig. 6:**
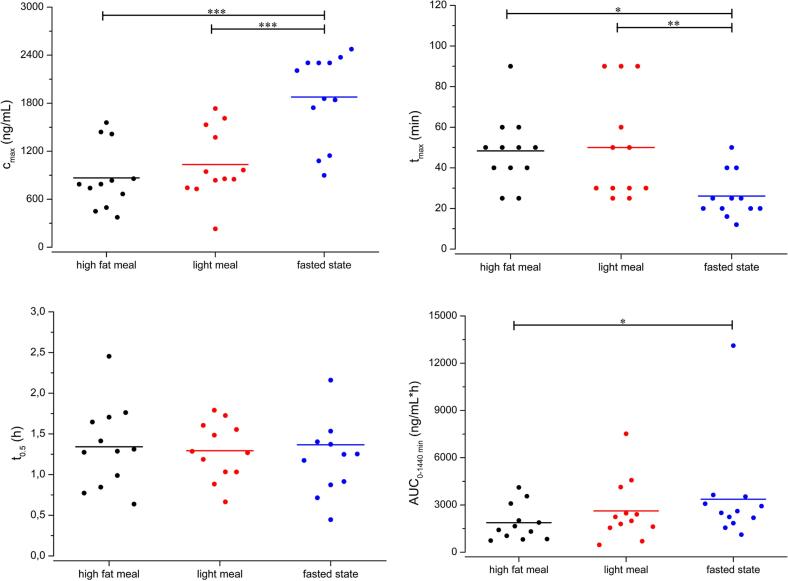
Statistical comparison of c_max_, t_max_, AUC_0__–__1440min,_ and t_0.5_ for methylliberine across study arms A-C. Differences in c_max_ were assessed using ANOVA followed by Tukey's *post hoc* test; AUC_0__–__1440min_, t0.5 and t_max_ was analysed using the Friedman test with Dunn's *post hoc* test. *p < 0.05; **p < 0.01; ***p < 0.001.

The statistical comparison of the three study arms (A-C) is shown in Fig. 5 for caffeine and Fig. 6 for methylliberine, including statistical evaluation of t_max_, c_max_, AUC_0__–__1440min_, and t_0,5_ across the three study arms. Statistically significant decreases in c_max_ and prolongations of t_max_ were observed for both caffeine and methylliberine in the fed state study trials when compared with the fasted study arm. No statistically significant differences were detected for either analyte with respect to the elimination half-life (t_0,5_). It is noteworthy that the AUC_0__–__1440min_ of caffeine did not demonstrate a statistically significant difference across the study arms A-C. However, for methylliberine, a statistically significant difference was observed between study arm A (high fat meal) and study arm C (fasted administration).

## 4 Discussion

In the present study, caffeine and methylliberine were quantified in saliva following oral administration as a rapidly releasing compression coated tablet. The absorption characteristics of the novel salivary marker methylliberine were compared with the absorption and systemic exposure of the well-established marker caffeine under different prandial conditions, including administration after a standardized high fat meal and a light meal, as well as under fasted conditions. Salivary tracer approaches represent a practical and non-invasive alternative to plasma based pharmacokinetic investigations, allowing frequent sampling with low burden for study participants and simplified study logistics. In this context, caffeine has been widely applied as a salivary marker due to its robust pharmacokinetic behaviour, however, its use is associated with practical limitations such as prolonged abstinence requirements, potential baseline contamination, and long sampling periods required to capture total exposure (Sager et al., 2018; Grimm et al., 2023c). Methylliberine has recently been proposed as a novel salivary marker for gastric emptying under fasted conditions, offering potential advantages over caffeine, including a shorter elimination half-life and the absence of relevant dietary background concentrations (Wildgrube et al., 2025). Consequently, the main reason for conducting this study was to evaluate methylliberine under postprandial conditions.

The combined administration of methylliberine and caffeine within a single compression coated tablet allowed a direct evaluation of the postprandial behaviour of methylliberine in relation to an established reference marker under identical physiological conditions. Compression coated tablets allowed for reliable and reproducible administration, preventing early disintegration in the oral cavity and thereby ensuring that measured salivary concentrations reflected systemic exposure rather than oral contamination. Compared with the compression-coated formulation developed by Tzakri et al. and applied in previous studies, the tablets used in the present work contained a higher load of salivary marker substances (Tzakri et al., 2023; Tzakri et al., 2024; Sarwinska et al., 2025). Consequently, a slight prolongation of tablet disintegration and drug release was observed for the formulations investigated. This was considered acceptable within the context of the present research question. Furthermore, the compression-coated tablets showed a slightly delayed release of approximately 30 s after the various stability studies and storage conditions. This delay was considered acceptable, as more than 85% of the marker substances were still released within the first five minutes, ensuring sufficiently rapid drug release. Each compression coated tablet contained 100 mg of methylliberine and 25 mg of caffeine. Both substances are well tolerated at these dose levels and are suitable for administration to healthy volunteers (Scientific Opinion on the Safety of Caffeine, 2015; Mondal et al., 2022; VanDusseldorp et al., 2020).

In all study arms (A-C), measurable salivary concentrations of both methylliberine and caffeine were already detected in the first post dose samples at 4 min, indicating rapid disintegration of the compression coated tablets after gastric arrival. Nevertheless, a statistically significant shift of t_max_ towards later time points was observed under postprandial conditions compared with fasted administration. These differences in t_max_ between fed and fasted conditions were statistically significant for both caffeine and methylliberine. For caffeine, mean t_max_ increased from 29 ± 12 min in the fasted state (study arm C) to 65 ± 35 min after the light meal (B) and 69 ± 46 min following the FDA breakfast (A). A similar trend was observed for methylliberine, with t_max_ values of 26 ± 11 min (C), 50 ± 27 min (B), and 48 ± 17 min (A). The extent of the postprandial delay varied between individual participants, but a shift of t_max_ towards later time points was observed in the majority of subjects. Nevertheless, a rapid initial increase in salivary concentrations was consistently detected across all study arms, indicating that, despite delayed gastric emptying, an early fraction of the administered formulation reached the absorptive sites *via* the *Magenstrasse*, a phenomenon describing fast emptying pathways in the postprandial stomach (Großmann et al., 2025; Pal et al., 2007; Grimm et al., 2017). At the same time, the delayed t_max_ values observed under fed conditions are fully consistent with established postprandial gastric emptying possibilities for solid oral dosage forms (Talukder and Fassihi, 2004). Three scenarios for active substances released from a dosage form in the postprandial stomach were described by Koziolek et al. *(*Koziolek et al., *2016**)*. According to this concept, rapid disintegration of a dosage form in proximity to the *Magenstrasse* may result in immediate transfer of the drug into the duodenum, leading to a rapid rise in systemic concentrations (type I scenario). In contrast, deposition of the dosage form in the gastric fundus or its homogeneous distribution within the chyme may result in delayed gastric emptying, characterised by either a sudden increase in exposure after a lag time (type II) or a slow and prolonged increase in systemic concentrations over several hours (type III) (Koziolek et al., 2016). In the present study, the early appearance of caffeine and methylliberine in saliva suggests that a relevant proportion of the marker substances was indeed transported *via* the *Magenstrasse* shortly after administration, indicating that the intended type I emptying mechanism of the compression-coated tablets was at least partially achieved. However, the delayed t_max_ values observed during food intake suggest that this process was not quantitative. It is therefore likely that some of the released marker substances mixed with the gastric contents instead of being completely initially absorbed due to the *Magenstrasse*. This partial mixing with the gastric contents may therefore result in absorption patterns similar to type II or type III scenarios, even though the initial release of the marker occurred near the *Magenstrasse*.

Despite these similarities in postprandial absorption kinetics, the overall systemic exposure differed markedly between the two salivary markers. With regard to caffeine, total exposure (AUC_0__–__1440min_) remained similar across all study arms, indicating that the food intake affected the rate, but not the extent, of absorption. A slight reduction in caffeine AUC was observed in the high fat meal arm, however, this difference was not statistically significant. This apparent decrease may be attributed to the limitations of the sampling scheme, as the 24 h observation period may not have fully captured late phase concentrations approaching the lower limit of quantification (LLOQ). In contrast, methylliberine exhibits a short elimination half-life, allowing for precise characterization of total systemic exposure. In the present study, elimination half-lives were consistently below 1.5 h across all prandial states (study arm A: 1.34 ± 0.51 h; B: 1.29 ± 0.35 h; C: 1.36 ± 0.75 h), comparable to values reported in previous studies (Mondal et al., 2022; Wang et al., 2020). In contrast to caffeine, a decrease in methylliberine AUC was observed as the caloric content of the administered meal increased, suggesting a potential negative food effect on methylliberine absorption. The reduction in AUC reached statistical significance when comparing the standardized high fat meal with the fasted administration. Potential causes of food effects can be direct interactions or unknown respectively indirect interactions, also referred as specific or unspecific interactions (Koziolek et al., 2019). While interactions with individual dietary compounds cannot be entirely excluded, there is currently no experimental evidence to support such mechanisms for methylliberine. In the absence of any known food related interactions, the observed reduction in methylliberine exposure is therefore more likely to be caused by unknown food interactions. Potential mechanisms underlying an unspecific negative food effect on methylliberine exposure may include physiological changes (Koziolek et al., 2019; Koziolek et al., 2015b). For example, it is known that food intake alters gastrointestinal motility, luminal composition, and bile salt concentrations, which can affect the solubility, permeability, and intestinal transit of orally administered compounds (Koziolek et al., 2019; Kambayashi and Shirasaka, 2023; Vinarov et al., 2023). The initial invasion rate and the resulting contact time with potential absorption windows, as well as changes in blood flow and metabolism after food intake, have also been suggested as factors that reduce the systemic exposure of certain compounds (Fleisher et al., 1999; Rose et al., 2017; Davis, 2005). These effects may be particularly relevant for substances with a short elimination half-life, such as methylliberine. In addition to the reasons mentioned, the concentration gradient in the upper small intestine and the viscosity of the luminal contents are also important factors (Tanaka et al., 2015; Aungst, 2020; Reppas et al., 1998). These considerations remain hypothetical as the present study was not intended to clarify individual mechanistic contributions. Furthermore, although the study population included equal numbers of male and female participants, the limited sample size did not allow a robust assessment of potential sex related differences. Nevertheless, visual inspection of the individual pharmacokinetic data did not indicate obvious sex dependent trends. Information regarding menstrual cycle phase was not collected in the present study. However, oral contraceptive use was documented during participant screening, with three participants reporting its use. Due to the small number of female participants, no subgroup analysis was performed. As hormonal status can affect both salivary characteristics and gastrointestinal physiology, this is a possible source of variability to consider in future studies (Puskulian, 1972; Tenovuo et al., 1981). Due to the study design, it cannot be determined whether the observed reduction in exposure reflects impaired absorption or an accelerated early phase elimination process.

Taken together, the present findings demonstrate that methylliberine exhibits a prandial dependency with respect to systemic exposure. While methylliberine shows favourable properties as a salivary marker under fasted conditions, the observed reduction in AUC under fed conditions limits its applicability as a postprandial salivary marker, particularly for applications relying on AUC based comparisons or exposure ratios between different study arms. Importantly, however, the rapid appearance of methylliberine in saliva shortly after dosing was consistently observed across all prandial states and closely resembled the behaviour of caffeine. Consequently, labelling of oral dosage forms under postprandial conditions remains feasible, particularly for applications focusing on early *in vivo* events such as time points of release and release rates of dosage forms.

The results suggest that the pharmacokinetics of methylliberine in saliva provide a non-invasive experimental framework for investigating interactions between formulation properties and physiological changes caused by food. In this regard, methylliberine may represent a useful compound for future mechanistic studies investigating the origins of negative food effects on oral drug absorption. Since methylliberine exhibited a calorie dependent reduction in systemic exposure, it may serve as a sensitive compound for studying the influence of fed state physiology, such as meal composition, gastric emptying behaviour, and luminal conditions, on oral drug absorption. If the observed reduction in exposure is driven by unspecific physiological factors rather than substance specific interactions, methylliberine could help to explore formulation or dosing strategies aimed at mitigating such food induced changes.

## 5 Conclusion

In the present study, a decrease in the overall systemic exposure of methylliberine was observed with increasing caloric content of the administered meal. This reduction in AUC reached statistical significance when comparing administration after a standardized high fat meal with fasted administration, whereas no changes in exposure were detected for caffeine. The results indicated that methylliberine is more susceptible to fed state physiology than caffeine and can therefore only be used to a limited extent as a salivary marker under postprandial conditions. Consequently, methylliberine cannot be considered a direct replacement for caffeine in fed state salivary tracer studies addressing biopharmaceutical questions. Nevertheless, the labelling of a dosage form with methylliberine remains feasible.

The pronounced sensitivity of methylliberine to postprandial conditions highlights its potential value as a compound for mechanistic investigations into the formation of food related changes in oral drug absorption. Although the underlying mechanisms were not investigated in the present study, methylliberine may support the systematic exploration of formulation-related contributors to food effects and the evaluation of strategies aimed at mitigating such effects.

## CRediT authorship contribution statement

**Toni Wildgrube:** Writing – review & editing, Writing – original draft, Visualization, Methodology, Investigation, Formal analysis, Data curation. **Nikolaus Alexander Link:** Writing – review & editing, Formal analysis. **Benno Ritucci:** Writing – review & editing, Methodology, Formal analysis. **Michael Grimm:** Writing – review & editing, Project administration, Methodology, Conceptualization. **Stefan Engeli:** Writing – review & editing, Visualization, Project administration. **Werner Weitschies:** Writing – review & editing, Visualization, Supervision, Project administration, Methodology, Funding acquisition, Conceptualization. **Philipp Schick:** Writing – review & editing, Visualization, Project administration, Methodology.

## Informed Consent Statement

Written informed consent was obtained from all individuals who volunteered to participate in the study. While the collected data do not permit the identification of individual participants, explicit written consent for publication of the manuscript was obtained from all subjects.

## Institutional Review Board Statement

This study was conducted in accordance with the principles of the Declaration of Helsinki (Helsinki 2024) and received approval from the Ethics Committee of Greifswald University Medicine (internal reference number: BB 175/24, approved on December 03, 2024). The study has been registered in the German Clinical Trials Register under the ID: DRKS00035516.

## Funding

No external funding was received for this research.

## Declaration of competing interest

The authors declare that they have no known competing financial interests or personal relationships that could have appeared to influence the work reported in this paper.

## Data Availability

Data will be made available on request.
