# Supplementary material for: Applicability of methylliberine as a salivary marker under postprandial conditions
Source: Int J Pharm X. 2026 Jun 15;12:100584. doi: 10.1016/j.ijpx.2026.100584 (PMC13293696; doi:10.1016/j.ijpx.2026.100584)
Supplement: Supplementary file 1 — Supplementary material [file mmc1.docx]

● A (high fat meal), ● B (light meal), ● C (fasted state)

|  |  |  |
| --- | --- | --- |
|  |  |  |
|  |  |  |
|  |  |  |

**Figure S1**. Graphical representation of the individual profiles (P01-P12) of caffeine following the administration of a compression-coated tablet after FDA-breakfast (study arm A-black), light meal (study arm B-red), fasted state (study arm C-blue).

● A (high fat meal), ● B (light meal), ● C (fasted state)

|  |  |  |
| --- | --- | --- |
|  |  |  |
|  |  |  |
|  |  |  |

**Figure S2**. Graphical representation of the individual profiles (P01-P12) of methylliberine following the administration of a compression-coated tablet after FDA-breakfast (study arm A-black), light meal (study arm B-red), fasted state (study arm C-blue).
